# Pseudotumoral Anogenital Herpes

**DOI:** 10.4269/ajtmh.25-0508

**Published:** 2026-04-14

**Authors:** Mingjia Hu, Shengli Chen, Hong Liu

**Affiliations:** ^1^ Dermatology Hospital of Shandong First Medical University, Jinan, Shandong, China;; ^2^ Shandong Provincial Institute of Dermatology and Venereology, Shandong Academy of Medical Sciences, Jinan, Shandong, China

## INTRODUCTION

**Figure 1.**
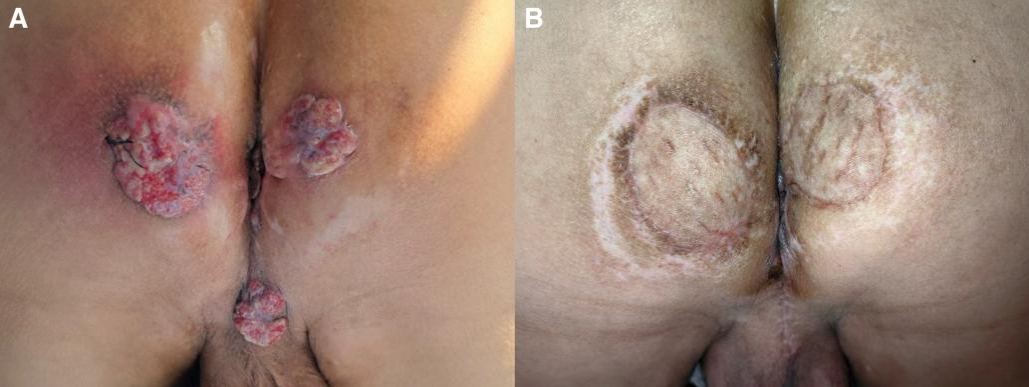
Cutaneous symptoms and histopathological features. (**A**) Perianal verrucous masses associated with erosion and ulceration. (**B**) Four months post-surgery, showing no signs of proliferative recurrence.

**Figure 2.**
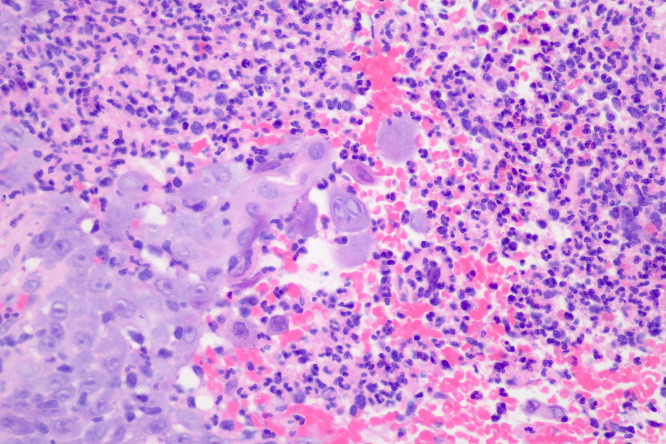
Histopathology reveals balloon-like cells and multinucleated giant cells, accompanied by extensive infiltration of lymphocytes and plasma cells (original magnification × 200).

A 77-year-old man presented with a 4-year history of exophytic hypertrophic verrucous lesions involving the perineal and gluteal regions, accompanied by erosion and ulceration, without associated pain or pruritus. Initially, the condition manifested as a flat plaque erosion and exudation, evolving into verrucous masses marked by localized erosion and ulceration (Figure 1A). The patient reported unprotected heterosexual intercourse with an extramarital partner within 1 year prior to symptom onset. He denied any prior history of genital herpes or other sexually transmitted infections. Histopathology of the gluteal lesion demonstrated keratinocyte ballooning degeneration and multinucleated giant cells, accompanied by dense infiltration of lymphocytes and plasma cells (Figure 2). HIV antigen–antibody and TPPA tests were negative; the CD4^+^ T-cell count was mildly reduced at 464 cells/mL (reference range: 550–1440). Polymerase chain reaction, clinical metagenomic next-generation sequencing, and type-specific IgG serology confirmed herpes simplex virus (HSV)-2 infection. Due to the presence of a large hypertrophic exophytic lesion, therapeutic surgical excision of the scrotal lesion was performed, with histopathological evaluation at multiple depths. The findings were consistent with the previously established diagnosis of herpetic infection. Oral valaciclovir (0.5 g twice daily) was initiated. Concurrently, limited local debulking measures were performed for the gluteal lesions. Given the substantial tension and extensive involvement of the gluteal skin, complete surgical excision would have required complex reconstruction and skin grafting. After discussion with the patient, a conservative approach was adopted, including gentle abrasion and a brief course of photodynamic therapy. Partial flattening of the verrucous lesions was observed. However, the patient discontinued photodynamic therapy due to pain intolerance and subsequently ceased valaciclovir. Lesion regrowth accompanied by surface ulceration and exudation was subsequently noted. Consequently, surgical excision and skin grafting were performed, and valaciclovir (0.5 g twice daily) was maintained. No recurrence was observed at the 6-month follow-up (Figure 1B). Pseudotumoral anogenital herpes has been reported as a rare and often misdiagnosed variant of genital herpes, more commonly described in immunocompromised individuals.^1^ It may present with atypical nodular, tumoral, or verrucoid growth patterns.^2^^–^^4^ Notably, in such cases, superficial biopsy specimens may be nonrepresentative and potentially misleading, sometimes delaying accurate diagnosis.^2^ In our patient, HIV testing was negative, and no secondary causes of immunodeficiency were identified. The mildly reduced CD4^+^ T-cell count may reflect age-related immunosenescence, which could contribute to impaired viral control.^5^ In addition, the perineal and gluteal location of the lesions—areas prone to chronic friction and moisture—may have promoted persistent inflammation and hypertrophic proliferation.
